# Liver protective effect of chloroform extract of *Bauhinia purpurea* leaves is attributed partly to its antioxidant action and the presence of flavonoids

**DOI:** 10.1080/13880209.2023.2241510

**Published:** 2023-08-09

**Authors:** Zainul Amiruddin Zakaria, Adibah Sahmat, Azfar Hizami Azmi, Amal Syahirah Nur Zainol, Maizatul Hasyima Omar, Tavamani Balan, Arifah Abdul Kadir, Syahriel Abdullah, Roro Azizah, Lilis Sulistyorini

**Affiliations:** a Borneo Research on Algesia, Inflammation and Neurodegeneration (BRAIN) Group, Faculty of Medicine and Health Sciences, Universiti Malaysia Sabah, Jalan UMS, Sabah, Malaysia; b Department of Environmental Health, Faculty of Public Health, Campus C Universitas Airlangga, Jalan Mulyorejo Surabaya, East Java, Indonesia; c Department of Biomedical Sciences, Faculty of Medicine and Health Sciences, Universiti Putra Malaysia, Selangor, Malaysia; d Herbal Medicine Research Centre, Institute for Medical Research, National Institutes of Health, Shah Alam, Malaysia; e Faculty of Pharmacy and Health Sciences, Universiti Kuala Lumpur Royal College of Medicine Perak, Ipoh, Perak, Malaysia; f Department of Veterinary Pre-clinical Sciences, Faculty of Veterinary Medicine, Universiti Putra Malaysia, Selangor, Malaysia; g Institute of Tropical Biology and Conservation, Universiti Malaysia Sabah, Jalan UMS, Sabah, Malaysia

**Keywords:** Liver injury, paracetamol intoxication, hepatoprotection, non-free radical scavenging, endogenous antioxidant enzymes system

## Abstract

**Context:**

*Bauhinia purpurea* L. (Fabaceae) is used in the Ayurvedic system to treat various oxidative-related ailments (e.g., wounds, ulcers etc.). Therefore, it is believed that the plant also has the potential to alleviate oxidative-related liver damage.

**Objective:**

This study elucidates the hepatoprotective activity of chloroform extract of *B. purpurea* leaves (CEBP) in paracetamol (PCM)-induced liver injury (PILI) rats.

**Materials and methods:**

Male Sprague-Dawley rats (*n* = 6) were pre-treated once daily (p.o.) with CEBP (50–500 mg/kg) for seven consecutive days before being administered (p.o.) a hepatotoxic agent, 3 g/kg PCM. Liver enzyme levels were determined from the collected blood, while the collected liver was used to determine the activity of endogenous antioxidant enzymes and for histopathological examination. CEBP was also subjected to radical scavenging assays and phytochemical analysis.

**Results:**

CEBP significantly (*p* < 0.05) reversed the toxic effect of PCM by increasing the serum levels of AST and ALT, and the activity of endogenous catalase (CAT) and superoxide dismutase (SOD) while reducing the liver weight/body weight (LW/BW) ratio. Other than low TPC value and radical scavenging activity, CEBP had a high antioxidant capacity when evaluated using the oxygen radical absorbance capacity (ORAC) assay. UHPLC-ESI-MS analysis of CEBP showed the presence of flavonoids.

**Discussion and conclusions:**

CEBP exerts its hepatoprotective activity through a non-free radical scavenging pathway that involves activation of the endogenous enzymatic antioxidant defense system. Further study is needed to identify the responsible bioactive compounds before the plant can be developed as a future alternative hepatoprotective medicament for clinical use.

## Introduction

Drug-induced liver injury (DILI) is one of the most common causes of acute liver injury in the United States (Kullak-Ublick et al. 2017). According to Garcia-Cortes et al. (2020), the estimated annual incidence rate of DILI is approximately 1.3–19.1 per 100,000 population. Paracetamol (PCM) is dose-dependently liver toxic in overdose, whether intentional or unintentional, and is one of the most commonly studied hepatotoxic drugs. The toxicity of PCM is attributed to its metabolization by CYP2E1 into excessive *N*-acetyl-*p*-benzoquinone imines (NAPQIs), which are free radicals that bind covalently to cellular nucleophiles (e.g., DNA, RNA and proteins), resulting in hepatocyte damage and death through a complex process that reduces the level of the endogenous antioxidant system.

NAPQI toxicity was the trigger for the search and development of the currently used antidote, *N*-acetylcysteine (NAC), which has been the only drug of choice for the treatment of PCM-induced hepatotoxicity for several decades. This antidote acts as a precursor or to replenish hepatic GSH, the major endogenous nucleophilic peptide that contributes to the neutralization of NAPQI (Aldini et al. 2018). Other potentials of NAC are also described elsewhere (Zhang et al. 2018; Šalamon et al. 2019). In parallel with the reports of NAC efficacy in the treatment of PCM-induced hepatotoxicity as described above, there are predictions that the global market for NAC will increase over the next five years (Šalamon et al. 2019). Despite the tremendous progress in the field of drug discovery, the increase in the global market for NAC is hampered by the fact that none of the modern hepatoprotective drugs on the market are able to completely avert or treat drug-induced liver injury (Mondal et al. 2020).

To avoid a global focus on NAC as the sole agent to treat liver injury, especially DILI, researchers have proposed an innovative approach of integrated drug discovery, carefully combining the potencies of traditional medicine with the modern concept of therapeutic evaluation of herbal products to search for new/alternative hepatoprotective lead candidates (Rakib et al. 2020). Considering that oxidative stress plays a crucial role in the development of liver injury, it is believed that plants with high antioxidant activity may have remarkable hepatoprotective effects (Lawal et al. 2017). One of the plants reported to have a significant antioxidant activity is *Bauhinia purpurea* L. (Fabaceae). *B. purpurea*, known as ‘pokok tapak kuda’ to the Malays, is native to southern China and India but has no documented medicinal value in Malaysian folk medicine. Traditional uses of *B. purpurea* in Ayurvedic system include treatment of wounds, ulcers, fever, gastric tumors, diarrhea, and glandular swellings (Kumar et al. 2019). Scientific studies have already revealed various pharmacological potentials of *B. purpurea* leaves (Pettit et al. 2006; Boonphong et al. 2007; Lakshmi et al. 2009; Ananth et al. 2010; Negi et al. 2012; Nafees et al. 2013; Yahya et al. 2013; Rana et al. 2016; Kumar et al. 2019), including antioxidant and hepatoprotective activities.

Although the methanol extract from the leaves of *B. purpurea* has been shown to exert hepatoprotective activity, the polarity of the hepatoprotective bioactive compounds could not be postulated because the solvent used is considered to be an intermediate solvent that dissolves polar (water-soluble) and nonpolar (lipid-soluble) groups (Yahya et al. 2013). The interaction between these two classes of compounds is expected to lead to the observed hepatoprotective activity. In our attempt to better understand the possible mechanisms of hepatoprotective action modulated by the lipid-soluble bioactive compounds of *B. purpurea* leaves, specifically, the chloroform extract was prepared and subjected to PCM-induced liver injury in a rat model. In addition, the extract was also evaluated for its antioxidant potential while its phytoconstituents were analyzed using HPLC and UHPLC-ESI-MS methods. In the present study, the hepatoprotective profile of CEBP was determined using the standardized *in vivo* PILI model and, the possible mechanisms of action and phytoconstituents involved were elucidated.

## Materials and methods

### Chemicals

Chloroform (99.8%; high purity; stabilized with amylene) was purchased from Fisher Scientific (Loughborough, UK). Silymarin (≥30% silybin; HPLC grade) and PCM (≥99.0%) were purchased from Sigma-Aldrich (MO, USA). All other chemicals and reagents used were of analytical grade.

### Collection of plant materials

The leaves of *B. purpurea* were collected between September and October 2020 near Universiti Putra Malaysia (UPM), Serdang, Selangor and compared with the specimen (voucher no.: SK 1985/11–20) in the herbarium of the Institute of Bioscience, UPM. *B. purpurea* L. was previously identified by Dr. Shamsul Khamis, a certified botanist of the herbarium.

### Preparation of chloroform extract of B. purpurea leaves

The leaves of *B. purpurea* were air-dried at room temperature in the shade for 2 weeks and then the stems were removed. The dried leaves were ground into powder using a sterile electric grinder. About 1 kg of the powdered leaves was mixed with chloroform at a ratio of 1:20 (w/v) and the mixture was stirred at room temperature for 72 h. The supernatant was filtered first using absorbent cotton and then with filter paper (Whatman No.1) to remove solid plant debris. The chloroform filtrates were concentrated by evaporation under reduced pressure at 40 °C to obtain the dried chloroform extract of *B. purpurea* (CEBP). The dried CEBP was then subjected to phytochemical screening, determination of total phenolic content, and UHPLC-ESI-MS analysis. In addition, the extract was tested for antioxidant and hepatoprotective activities.

### Analysis of phytoconstituents of CEBP

#### Phytochemical screening of CEBP

Phytochemical screening of CEBP was performed according to the method described in detail by Ismail Suhaimy et al. (2017) to determine the presence of alkaloids, flavonoids, saponins, tannins, triterpenes and steroids.

#### UHPLC-ESI-MS analysis

The UHPLC-ESI-MS system was used to analyze the phytoconstituents of CEBP. It consists of a Dionex 3000 UHPLC system (Thermo Fisher Scientific, USA) coupled to a linear ion trap- Orbitrap hybrid mass spectrometer (Q Exactive; Thermo Fisher Scientific, USA) equipped with an electrospray ionization (ESI) source. The detailed conditions for each compartment of the system used to analyze the sample have been described in detail elsewhere (Ismail Suhaimy et al. 2017).

### Antioxidant studies of CEBP

#### Total phenolic content

Total phenolic content (TPC) of CEBP was determined according to the slightly adapted method of Singleton and Rossi (1965), which was described in detail by Ismail Suhaimy et al. (2017).

#### Diphenylpicrylhydrazyl radical scavenging assay

The diphenylpicrylhydrazyl (DPPH) radical scavenging assay was performed based on the modified procedure of Blois (1958), which has been described in detail elsewhere (Ismail Suhaimy et al. 2017). Green tea (*Camellia sinensis* (L.) Kuntze) with an IC_50_ of 20.81 μg/mL was used as a reference standard.

#### Superoxide anion radical scavenging assay

The superoxide anion (SOA) radical scavenging assay was performed based on the modified method of Chang et al. (1996) which was described in detail by Ismail Suhaimy et al. (2017). Ascorbic acid with an IC_50_ of 97.53 μg/mL was used as a reference standard.

#### Oxygen radical absorbance capacity test

The oxygen radical absorbance capacity (ORAC) assay was performed according to the modified procedure of Huang et al. (2002), which was described in detail by Ismail Suhaimy et al. (2017). Trolox with an IC_50_ of 10.83 µM was used as a reference standard.

#### Experimental animals

This study was conducted in accordance with the guidelines of the Declaration of Helsinki, and ethical approval was obtained from the Institutional Animal Care and Use Committee (IACUC), FMHS, UPM (Ref. No: UPM/FPSK/PADS/BR-UUH/00506) using adult male Sprague-Dawley rats (180–220 g; 8 to 10 weeks old). The procedure for acclimatization and feeding of the animals under standard husbandry conditions has been described in detail elsewhere (Ismail Suhaimy et al. 2017). Prior to the experiments, the animals were given access to water for only 48 h to ensure complete gastric emptying before oral administration of the extract. Gastric emptying has been reported to help increase the uptake of the extract into the body system (Nowland et al. 2011; Omachi et al. 2019).

### Hepatoprotective assay

#### PCM-induced hepatotoxicity test

The extract was diluted in 8% Tween 80 to produce the extract at dosages of 50, 250, and 500 mg/kg, which was then assayed for hepatoprotective activity. Fasting rats (*n* = 6) were divided into six groups and each group received (p.o.) 10% DMSO (normal; Group 1), 10% DMSO (negative; Group 2), 50 mg/kg NAC (positive; Group 3), 50 mg/kg CEBP (treatment; Group 4), 250 mg/kg CEBP (treatment; Group 5) or 500 mg/kg CEBP (treatment; Group 6) daily for 7 consecutive days. The test solutions were administered orally to mimic the traditional use of the plant extract, while the CEBP doses used were selected based on the previous acute toxicity study of CEBP (Zakaria et al. 2011) and the dose range suggested by Schmeda-Hirschmann and Yesilada (2005). PCM (3 g/kg) was administered 3 h after the last administration of the respective test solution on day 7. Forty-eight hours after administration of PCM, rats were anesthetized with a combination of ketamine (50 mg/kg) and xylazine (5 mg/kg) to allow blood collection *via* cardiac puncture for biochemical analyzes before being sacrificed by cervical dislocation to collect the liver for histopathological studies and preparation of liver homogenates.

#### Biochemical analyses

The collected blood was processed to obtain the serum, which was then subjected to the determination of level of serum liver enzymes [e.g., aspartate aminotransferase (AST), alanine aminotransferase (ALT) and alkaline phosphate (ALP)], total and direct bilirubin, and total protein (Ismail Suhaimy et al. 2017).

#### Histopathological study

The collected liver tissue from each group was immediately washed and processed to prepare H&E-stained liver sections (5–6 μm thickness), which were then examined for histopathological changes and scored according to the severity of liver injury as described by El-Beshbishy et al. (2010).

### Assessment of the liver’s endogenous antioxidant enzymes activity

#### Preparation of liver homogenates

Each liver tissue (100 mg) was minced in 1 mL cold PBS buffer and homogenized using a steel homogenizer before the homogenate was centrifuged (4,000 rpm; 25 min; 4 °C) using Sorvall™ Legend™ Micro 17 R microcentrifuge (Thermo Fisher Scientific). The supernatant was collected and then the activity of antioxidant enzymes [superoxide dismutase (SOD) and catalase (CAT)] was determined using the Cayman Assay Kit (Cayman Chemical, USA) according to the manufacturer’s protocol.

#### Statistical analysis

Data were presented as mean ± SEM and analyzed using one-way ANOVA followed by the Dunnett’s *post hoc* test. *p*** **<** **0.05 was considered as significant.

## Results

### Phytoconstituents of CEBP

#### Phytoconstituents screened in CEBP

Qualitative phytochemical screening of CEBP revealed the presence of several classes of bioactive compounds namely flavonoids, triterpenes and steroids (Table 1).

**Table 1.** Qualitative Findings on the phytochemical constituents of CEBP.

Type of extract	Phytochemical constituents	Scoring of bioactive compounds
CEBP	Alkaloids	–
	Saponins	–
	Flavonoids	2+
	Tannins	–
	Triterpenes	3+
	Steroids	3+

Note that: –: Not detected; 1+: Weak colour; 2+: Mild colour; 3+: Strong colour.

#### Characterization of bioactive compounds from CEBP by UHPLC-ES-MS

**Figure 1.**
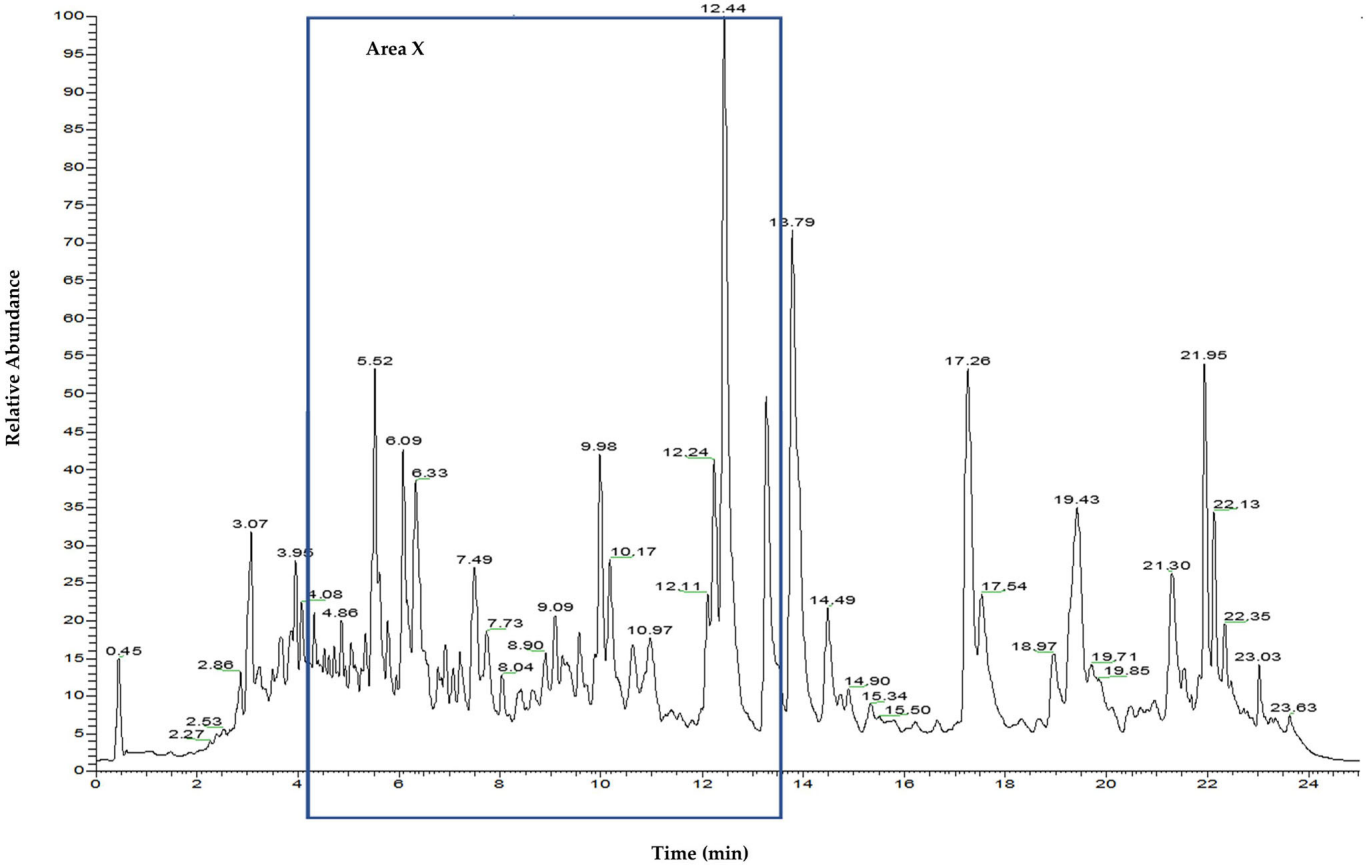
UHPLC-ESI-MS profile of CEBP showing various flavonoids and caffeic acid derivatives.

**Figure 2.**
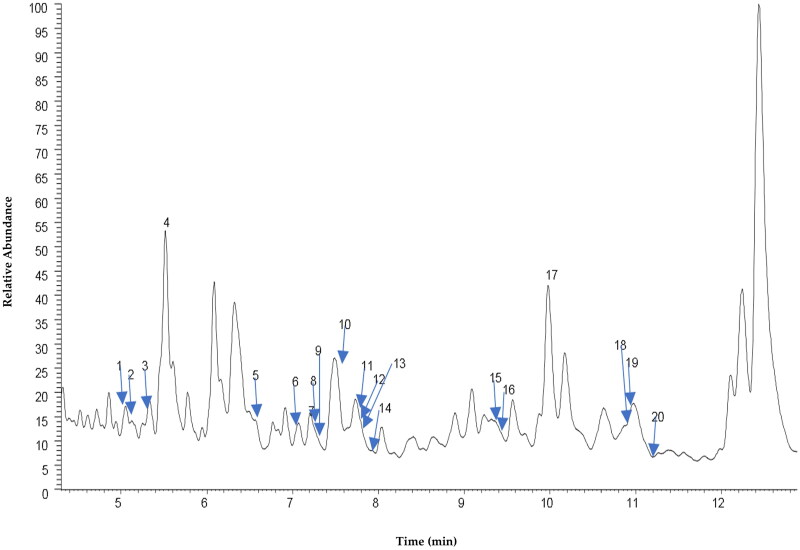
Total ion chromatography (TIC) UHPLC-ESI of *B. purpurea* leaves extract characterized by one main region (area X) with peaks mainly due to flavonoids.

Figure 1 shows the total ion chromatogram (TIC) of CEBP analyzed using the UHPLC-ESI-MS between 0 and 24 min. Figure 2 shows the TIC of CEBP between 4 and 12 min, indicating the presence of various compounds classified as flavonoids and caffeic acid derivatives. The compounds identified experimentally are listed in Table 2 with their retention time, molecular formula and calculated mass (m/z), also taking into account the data reported in the literature.

**Table 2.** Peak assignment of metabolites in CEBP extract using UHPLC-ESI-MS in negative ionization mode.

Peak No	t_R_ (min	Tentative assignment	Molecular formula	[M-H]	Error (ppm)
1	5.16	Shikoniin	C_16_H_15_O_5_	287.09213	2.543
2	5.30	Naringenin	C_15_H_11_O_5_	271.06064	1.992
3	5.32	Luteolin	C_15_H_9_O_6_	285.04019	2.896
4	5.56	Isorhamnetin	C_16_H_11_O_7_	315.05142	4.732
5	6.45	Flavanone isomer i	C_15_H_11_O_3_	239.07097	2.925
6	7.05	5-Hydroxy-4′-7-dimethoxyflavanone	C_17_H_15_O_5_	299.09250	3.678
7	7.20	7,4′-Dihydroxy-5-methoxy-flavanone	C_16_H_13_O_5_	285.07660	2.982
8	7.25	Pinocembrin	C_15_H_11_O_4_	255.06604	3.351
9	7.3	Huaicarbon B	C_21_H_15_O_9_	411.07239	3.239
10	7.53	3,7-Dihydroxyflavone	C_15_H_9_O_4_	253.05042	3.496
11	7.76	7,8-Dihydroxyflavone	C_15_H_11_O_4_	255.06595	2.998
12	7.75	Pinostrobin	C_16_H_13_O_4_	269.08139	2.061
13	7.77	Diosmetin	C_16_H_11_O_6_	299.05579	2.593
14	7.98	Galangin	C_15_H_9_O_5_	269.04504	2.193
15	9.32	Dunnione	C_15_H_13_O3	241.08641	2.524
16	9.41	Flavanone isomer ii	C_15_H_11_O_3_	239.07104	3.218
17	9.97	5-Hydroxy-7,4′-dimethoxyflavone	C_17_H_13_O_5_	297.07660	3.265
18	10.89	3-Hydroxy-5,7,8-trimethoxyflavone	C_18_H_15_O_6_	327.08789	4.816
19	10.93	Caffeic acid phenetyl esther	C_17_H_15_O_4_	283.09708	9.000
20	11.2	5-Hydroxy-7,8,3′,4′-tetramethoxyflavone	C_19_H_17_O_7_	357.09860	4.819

### Antioxidant property and activity of CEBP

#### TPC value of CEBP

The TPC value of 200 µg/mL CEBP was about 34.1 ± 1.3 mg/100 g GAE, indicating that the extract contained very low levels of total phenolic compounds. Based on the consideration that the highest TPC value is reached when the value is more than 1000 mg GAE/100 g, it is plausible to assume that CEBP has a low TPC value.

#### Effect of CEBP on DPPH radical scavenging assay

At a concentration of 200 μg/mL, CEBP showed low radical scavenging activity (14.7 ± 0.1% inhibition) as measured by the DPPH assay. The IC_50_ value is not detectable because the percentage of inhibition measured was less than 50%.

#### Effect of CEBP on SOA radical scavenging assay

At a concentration of 200 μg/mL, CEBP also showed low radical scavenging activity (21.4 ± 3.0% inhibition) as measured by the SOA assay although the IC_50_ value is indeterminate because the recorded percentage of inhibition was less than 50%.

#### Effect of CEBP on ORAC assay

CEBP, at a concentration of 160 μg/mL, gave an ORAC value of about 22,900 μmol TE/100 g.

### Hepatoprotective effect of CEBP against PCM-induced liver injury

#### Effect of CEBP on body and liver weights of PCM-intoxicated rats

Table 3 shows the effect of CEBP on body and liver weights, and the ratio of liver to body (liver/body) weights of PCM-intoxicated rats. PCM intoxication (negative control group) resulted in a significant (*p* < 0.05) increase in liver but not body weight of rats compared to the normal control group; this indicates that successful liver injury was achieved and the application of 600 mg/kg PCM as an inducer of liver injury provided reliable data. Only rats pretreated with 500 mg/kg CEBP or 50 mg/kg NAC significantly (*p* < 0.05) reduced the toxic effect of PCM as indicated by a reduction in liver weight and liver/body weight ratio. The reduction in liver weight, which directly decreases the liver/body weight ratio, indicates that the extract at a dose of 500 mg/kg has a reliable hepatoprotective effect.

**Table 3.** Effect of CEBP on the body and liver weights, and liver/body weight ratio of PCM-intoxicated rats.

Treatment	Dose (mg/kg)	Body weight (BW) (g)	Liver weight (LW) (g)	LW/BW (%)
Normal	–	207.9 ± 4.7	6.2 ± 0.4	3.0
Negative	–	205.5 ± 8.3	8.8 ± 0.7^a^	4.3^a^
Positive	50	203.6 ± 2.7	7.1 ± 0.4^b^	3.5^b^
CEBP	50	200.7 ± 5.6	8.1 ± 0.9	4.0
	250	197.1 ± 0.7	7.8 ± 0.8	4.0
	500	198.2 ± 4.3	5.3 ± 0.2^b^	2.7^b^

Values are expressed as means ± SEM of six replicates.

^a^Data differed significantly (*p* < 0.05) when compared against the normal control group.

^b^Data differed significantly (*p* < 0.05) when compared against the negative control group.

#### Effect of CEBP pretreatment on the serum liver AST and ALT level of PCM intoxicated rats

The effect of CEBP on PCM-induced liver intoxication was evaluated by performing liver function test and the results are shown in Table 4. It was found that the serum levels of AST and ALT significantly (*p* < 0.05) increased in the PCM-intoxicated group compared to the normal group, indicating that liver enzymes leaked into the bloodstream due to PCM-induced liver injury. Pretreatment of PCM-intoxicated rats with CEBP at doses of 50, 250 or 500 mg/kg showed a significant (*p* < 0.05) reduction in serum levels of AST and ALT, indicating a protective effect of CEBP against the toxic effects of PCM. Interestingly, 50 mg/kg NAC (positive control) also reduced serum levels of both enzymes.

**Table 4.** Effect of CEBP on the serum level of AST and ALT of PCM-intoxicated rats.

Treatment	Dose (mg/kg)	ALT (U/L)	AST (U/L)
Normal	–	15.8 ± 2.9	95.1 ± 5.9
Negative	–	171.4 ± 14.2^a^	302.6 ± 38.3^a^
Positive	–	88.4 ± 19.5^b^	122.8 ± 22.9^b^
CEBP	50	161.9 ± 36.2	337.1 ± 17.7
	250	89.9 ± 29.9^b^	251.6 ± 37.1^b^
	500	55.7 ± 9.4^b^	154.7 ± 34.2^b^

Values are expressed as means ± SEM of six replicates.

^a^Data differed significantly (*p* < 0.05) when compared against the normal control group.

^b^Data differed significantly (*p* < 0.05) when compared against the negative control group.

#### Histopathological observations of the effect of CEBP pretreatment on the structure of rat’s liver intoxicated with PCM

**Figure 3.**
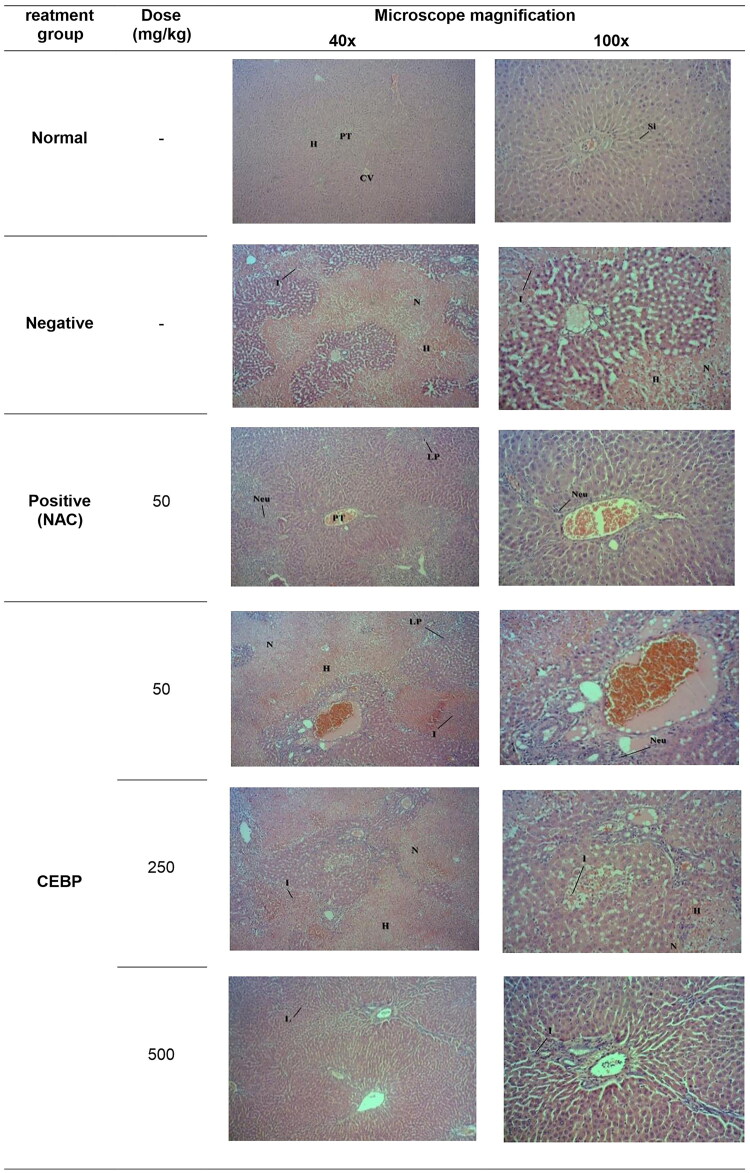
Histopathological studies of PCM-intoxicated liver tissue from rats pretreated with CEBP or NAC at 40x and 100x magnification. Liver tissue from the normal group showed normal liver architecture damaged by intoxication with 3 g/kg PCM as indicated by the apppearence of massive necrosis **(N)**, hemorrhage **(H)**, and inflammation **(I)**. pretreatment with 5 mg/kg NAC reversed the PCM induced toxicity in the liver tissue causing moderate **I** as evidenced by the presence of neutrophils **(Neu)** and lymphoplasmacytic **(LP)** cells, whereas the portal tract exhibited a mild **I** as indicated by the presence of scattered **Neu**. On the other hand, pretreatment with 50 mg/kg CEBP failed to reverse the effect of PCM as indicated by the presence of a severe **H** with an area of **N** with infiltration of **I** cells and the presence of **LP** cells. Pretreatment with 250 mg/kg CEBP caused mild **I** in liver tissue, as indicated by the presence of lymphocytic cells **(L)** without **N** and **H** domains, with portal tract showing the presence of scattered **I** cells in the liver parenchyma. Finally, pretreatment with 500 mg/kg CEBP successfully reversed the toxic effect of PCM on liver tissue, as indicated by the absence of **H**, **N,** and **I**, whereas the portal tract showed only a mild infiltration of **Neu** in the liver parenchyma.

To confirm the ability of PCM to induce liver injury and to verify the hepatoprotective potential of CEBP against the toxic effects of PCM, histopathological examination was performed and the results are shown in Figure 3. The normal group exhibited normal lobular architecture and normal hepatic cells characterized by the presence of well-preserved and intact cytoplasm, prominent nucleus, well-defined sinusoidal spaces and mostly visible veins. However, PCM-induced intoxication resulted in liver injury manifested by severe centrilobular necrosis, hemorrhage formation, presence of inflammatory exudates, infiltration of lymphocytes and steatosis. Pretreatment with 50 mg/kg NAC reversed the toxic effect of PCM, as evidenced by the presence of moderate inflammation caused by infiltration of neutrophils and lymphoplasmacytic cells with scattered neutrophils in the portal vein. On the other hand, pretreatment of PCM intoxicated rats with CEBP caused a remarkable reversal of the pathological changes observed in the negative control group, indicating the ability of the extract to attenuate PCM-induced intoxication. Table 5 shows the histopathological evaluation of liver tissues intoxicated with PCM after pretreatment with CEBP or NAC. As with NAC pretreatment, the extract also reduced the severe formation of necrosis, hemorrhage, and inflammation observed in the negative control group.

**Table 5.** Histopathological scoring of the PCM-intoxicated liver tissues following pretreatment with CEBP, at various doses, or NAC.

Treatment	Dose (mg/kg)	Steatosis	Necrosis	Inflammation	Haemorrhage
Normal	–	–	–	–	–
Negative	–	–	+++	+++	+++
Positive	50	–	+	+	+
CEBP	50	–	+++	+++	+++
	250	–	++	++	++
	500	–	–	+	+

The severity of various features of liver injury was evaluated based on those following scoring schemes: –: normal; +: mild effect; ++: moderate effect; +++: severe effect.

#### Effect of CEBP on the liver tissue’s endogenous antioxidant system

Table 6 shows the modulatory effect of CEBP on the endogenous antioxidant enzymes of the liver tissues of rats intoxicated with PCM. The liver tissue of PCM-intoxicated group (negative control) showed a significant (*p* < 0.05) decrease in the activities of CAT and SOD compared to the normal group. Only pretreatment with NAC or CEBP at doses of 250 and 500 mg/kg caused a significant (*p* < 0.05) reversal of PCM intoxication by increasing the activities of CAT and SOD compared to the negative control group.

**Table 6.** Modulatory effect of CEBP on the activity of liver’s endogenous antioxidant enzyme system, namely CAT and SOD, in PCM-induced liver intoxication in rats.

Treatment	Dose (mg/kg)	CAT (U/g tissue)	SOD (U/g tissue)
Normal	–	187.3 ± 3.2	137.3 ± 7.2
Negative	–	85.6 ± 4.7^a^	66.8 ± 11.3^a^
Positive	50	132.8 ± 6.1^ab^	119.5 ± 5.7^a,b^
CEBP	50	91.5 ± 9.4^a^	73.7 ± 6.7^a^
	250	144.2 ± 9.5^a,b^	105.4 ± 5.9^a,b^
	500	176.6 ± 7.7^a,b^	122.2 ± 8.1^a,b^

Values are expressed as means ± SEM of six replicates.

^a^Data differed significantly (*p* < 0.05) when compared against the normal control group.

^b^Data differed significantly (*p* < 0.05) when compared against the negative control group.

## Discussion

Despite its good safety profile, accidental or incidental PCM overdose, especially in children, can lead to severe hepatic necrosis, which can result in fatal liver failure if not treated appropriately (Caparrotta et al. 2018). Treatment of PCM overdose is based on several factors, including early intravenous treatment with the sole antidote, NAC. Although NAC is effective for treating PCM-induced liver injury (PILI) when used early enough, its narrow therapeutic window limits its use. Therefore, it is necessary to find other alternative agents for the treatment of PILI rather than relying only on NAC. One of the sources of new hepatoprotective agents is plants, and several plants have been reported to exert hepatoprotective effects (Ugwu and Suru 2021). Although our group has previously reported the hepatoprotective activity of methanolic extract of B. purpurea leaves (MEBP) against PCM-induced liver intoxication in rats, methanol tends to extract both water- and lipid-soluble bioactive compounds, suggesting that the interaction between these types of compounds may lead to the observed hepatoprotective activity of MEBP (Yahya et al. 2013).

In an attempt to investigate the hepatoprotective potential of the lipid-soluble or hydrophobic bioactive compounds present in the leaves of B. purpurea, the chloroform extract (CEBP) was used in this study. Based on the results obtained, several summaries could be made about CEBP including: (i) CEBP contained only flavonoids, triterpenes and steroids; (ii) analysis of UHPLC-ESI-MS showed the presence of numerous flavonoid-based bioactive compounds, some of which constituted the phytoconstituents of CEBP; (iii) CEBP had a low TPC value and exerted a very low radical scavenging effect when measured by DPPH or SOA radical scavenging assay but showed a reasonable antioxidant capacity evaluated by ORAC assay, and; (iv) CEBP showed a hepatoprotective effect at 250 and 500 mg/kg, with a remarkable protective effect only at the latter dose, as confirmed by serum liver enzyme level, LW/BW ratio, histopathological examination and evaluation, and endogenous liver enzyme system activity.

Flavonoids were detected in phytochemical screening, but the TPC content of CEBP was significantly low. It is known that phenolic compounds are classified into numerous subclasses (i.e., phenolic acids, tannins, flavonoids, curcuminoids, coumarins, quinones, lignans, and stilbenes) (Gan et al. 2019). Therefore, the low TPC value could be due to the presence of a small amount of the above-mentioned non-flavonoid compounds, with the exception of tannins, which were not present in the qualitative phytochemical screening analysis. Moreover, this result also suggests that the mechanisms of antioxidant modulation by the lipid-soluble compounds are different but still contribute to the antioxidant and hepatoprotective activities of MEBP (Yahya et al. 2013).

It is well known that phenolic compounds possess redox properties responsible for antioxidant activity due to hydroxyl groups, which are good electron donors; thus, accelerating the degradation of free radicals (Platzer et al. 2022). Several reports have shown the relationship between TPC content and radical scavenging strength (Aryal et al. 2019), and in agreement with these reports, the low TPC value in CEBP is hypothesized to be the cause of the low radical scavenging activity of CEBP in both DPPH- and SOA-radical scavenging assays. In addition to the radical scavenging effect, some of the phenolic compounds have the ability to stimulate cells to synthesize endogenous antioxidant molecules (Barraza-Garza et al. 2020), while others have been found to scavenge oxygen, deactivate metals, or degrade peroxides in biological systems and avert the burden of oxidative diseases (Babbar et al. 2015). The different types of antioxidants described above suggests that phenolic compounds have multiple capabilities to exhibit antioxidant activity; this highlights the appropriate antioxidant capacity of CEBP when assessed by the ORAC assay.

As previously reported, the present study also showed that oral administration of 600 mg/kg PCM induced liver injury in rats, as evidenced by increased LW/BW ratio, elevated serum levels of AST and ALT, and severe damage to liver architecture as indicated by the presence of necrosis, hemorrhage and inflammation on histopathologic examination of intoxicated liver tissue compared to normal liver tissue. Pretreatment with CEBP reversed the toxic effect of PCM and returned the LW/BW ratio and, serum levels of AST and ALT to normal, while protecting the liver architecture from severe damage. These results were clearly observed only at the doses of 250 and 500 mg/kg CEBP compared to methanol extract of B. purpurea leaves, which improved PILI in the dose range of 50–500 mg/kg (Yahya et al. 2013). Given this comparison, it is reason able to assume that MEBP attenuates the effect of PILI to a greater extent compared to CEBP, which may be related to the fact that MEBP has a high TPC value (≈1194.4 mg GAE/100 g) and high radical scavenging activity in the DPPH radical scavenging assay (≈60% inhibition). Despite the low TPC value and the lack of radical scavenging effect, the ability of CEBP to attenuate PILI could possibly be due to three factors, namely: (i) the presence of phenolic compounds with the ability to activate antioxidant activity *via* different mechanisms of action, as previously described (Babbar et al. 2015; Barraza-Garza et al. 2020); (ii) the presence of triterpenes with hepatoprotective activity (Xu et al. 2018; Yin et al. 2019), and; (iii) the involvement of different cellular processes in modulating PCM-induced liver injury, as briefly described below (Yan et al. 2018). Although mitochondrial oxidative stress is considered the major cellular event in PILI, it has been recognized that numerous other cellular processes contribute to the pathogenesis of PILI, including phase I/phase II metabolism, microcirculatory dysfunction, liver regeneration, endoplasmic reticulum stress, autophagy and sterile inflammation (Yan et al. 2018). It is also worth-noting that there is a report on the ability of phytosterols to exacerbate liver injury associated with parenteral nutrition (Hukkinen et al. 2017). Although the effect of phytosterols on PILI has never been reported to our knowledge, it is possible that the presence of a high content of steroids in CEBP could contribute to the extract being less effective than MEBP in attenuating PILI as mentioned previously.

Despite the steady increase in the number of patients with liver damage, currently available treatments still produce unsatisfactory results. Therefore, scientists are striving to find new or alternative treatments to cure liver damage, and one of the sources of medicines to treat liver disease is plants. Medicinal plants have been studied scientifically, and a growing body of evidence suggests that these medicinal plants may be developed as hepatoprotective drugs in the future. It is important to emphasizcomparee that the use of medicinal plants as hepatoprotective agents depends on our knowledge of the individual phytoconstituents in each plant and their interactions with each other. Although flavonoids, triterpenes and steroids were detected in CEBP, these compounds contributed less to the radical scavenging capacity of the extract but contributed very much to the ORAC-measured antioxidant capacity of CEBP. Since most flavonoids previously reported showed radical scavenging activity against both radical scavenging assays used in this study (Kaurinovic and Vastag 2019), it is plausible that the flavonoids detected in CEBP use other types of antioxidant mechanisms as the main pathway instead of the radical scavenging mode (Babbar et al. 2015; Barraza-Garza et al. 2020). Apart from this, triterpenes are also known to possess antioxidant and hepatoprotective activities, the former of which may also occur *via* non-radical scavenging-mediated pathways; thus, may play a role in the observed hepatoprotective activity of CEBP. Among the compounds detected using UHPLC-ESI-MS, shikonin (Guo et al. 2019), naringenin (Ahmed et al. 2019), and galangin (Tsai et al. 2015), were previously reported to exert hepatoprotective activity against PILI. On the other hand, compounds such as shikonin (Zuo et al. 2018), naringenin (Rashmi et al. 2018), galangin (Ouyang et al. 2018), diosmetin (Yang et al. 2017), and pinostrobin and pinocembrin (Wahyuni et al. 2018) were reported to possess radical scavenging activity. The presence of various radical scavenging compounds does not justify the low radical scavenging activity of CEBP. One possible explanation is that a negative synergistic effect between some of the compounds present in CEBP, especially flavonoids, leads to the loss of radical scavenging activity of the extract. This explanation is supported by the results of Hidalgo et al. (2010) who reported that the combination of certain flavonoids promoted a positive synergistic effect, whereas other combinations promoted negative synergistic/antagonistic effects in the DPPH scavenging assay as well as in the FRAP assay.

## Conclusions

CEBP contains lipid-soluble hepatoprotective compounds with high antioxidant capacity that act primarily through non-radical scavenging-mediated mechanisms; therefore, they are thought to contribute to MEBP-induced hepatoprotective activity. Further studies are planned to potentially isolate new bioactive compounds with hepatoprotective activity from CEBP for future drug development.

## Data Availability

The datasets used and/or analyzed in this study are available from the corresponding author upon reasonable request.
